# Nutrition knowledge and health vulnerability of mothers of pre-school children in north-central, Nigeria

**DOI:** 10.1371/journal.pone.0292252

**Published:** 2024-01-31

**Authors:** Bosede Alice Omachi, Annette van Onselen, Unathi Kolanisi

**Affiliations:** 1 University of KwaZulu-Natal, Pietermaritzburg, KwaZulu-Natal, South Africa; 2 University of South Africa, Florida, South Africa; 3 University of Zululand, eMpageni, Kwazulu-Natal, South Africa; Federal University of Agriculture Abeokuta, NIGERIA

## Abstract

**Objective:**

This study explores the contribution of nutrition knowledge to the health status of pre-school children’s mothers in Niger State, North-Central, Nigeria.

**Design:**

The study is a descriptive cross-sectional design using a quantitative data collection method.

**Setting/participants:**

A multi-stage sampling technique was used to recruit 450 mothers of pre-school children across Niger state, Nigeria. Chi-square and linear regression were used to test the level of statistical significance (at p < 0.05). Socioeconomic and demographic information, anthropometric indices and nutrition knowledge were obtained using semi-structured questionnaires. Feeding patterns were also assessed using a qualitative 7-day dietary recall.

**Result:**

The results showed that the majority (63.8%) of the mothers were within 26–35 years, and more than half (51.6%) of the mothers lacked knowledge of a “balanced diet”. Fruits, vegetables, and dairy products were the least consumed food group among the mothers (7.1% and 9.1%, respectively). Place of residence, occupation, and method of waste disposal were significantly associated with maternal minimum dietary diversity adequacy (*p*<0.05). Over half (57.6%) of the mothers were within the normal BMI range, and the mean waist/hip ratio was 0.82± 0.08. Social media/online was the most (36.4%) explored source of nutrition information among the mothers. This study shows no significant association between nutrition knowledge and adequacy of minimum dietary diversity among the mothers of preschool children (*p* = 0.09, χ^2^ = 13.682).

**Conclusion:**

Dietary diversity among mothers was associated with the socioeconomic status and BMI of the mothers, which were strong determinants of meal quality and health outcomes in Nigeria and other developing countries experiencing food insecurity.

## 1. Introduction

Maternal nutrition refers to the nutritional needs of women throughout their reproductive lives. It is a potential tool for preventing the under or over-consumption of nutrients that could lead to diet-related non-communicable diseases among women, children, and the elderly [1]. Food is an essential determinant of an individual or household’s nutritional status. Hence, knowledge about dietary needs among mothers is essential for good health and overall nutritional status especially when available resources are limited. Often mothers do not know the importance of varieties and how to balance the food plate/diet in the correct proportion needed to meet their dietary needs [1].

Maternal nutrition knowledge is essential for optimal health outcomes among mothers and their households because food acquisition, meal planning, and preparation is usually the responsibility of women in most households in Africa [2, 3]. However, poor nutrition knowledge and nutrient intake among women of childbearing age result in low maternal pre-pregnancy BMI, anaemia-induced mortality, increased susceptibility to infections and poor pregnancy outcomes like low birth weight (LBW) and failure to thrive in infants [3, 4].

Women of childbearing age are a significant proportion of nutritionally disadvantaged groups globally because of the extra-nutrient demand in response to physiological changes [4]. Poor feeding patterns, socioeconomic status, suboptimal food systems and environment, inadequate dietary diversity, infections and short interpregnancy spacing often aggravate the risk of malnutrition and mortality among this vulnerable group [5].

Food choices and consumption patterns strongly predict overall health, wellness, and pregnancy outcomes among women of reproductive age [6]. The impact of poor dietary intake among mothers is severe for both the mother and child, resulting in increased maternal and child morbidity and mortality [7].

Poor feeding habits among mothers have been linked to urbanisation and nutrition transitioning which is common in developing countries like Nigeria. It has increased the consumption of nutrient-poor foods and beverages nationwide [8]. Over-consumption of energy-dense, ultra-processed food also referred to as ‘full but empty plate’ among mothers has been associated with an increased risk of ischaemic heart disease, stroke, atherosclerosis, insulin resistance diabetes, chronic kidney disease, osteoporosis, some cancers, and excessive weight gain [3, 9].

The outcomes of the poor dietary pattern are all forms of malnutrition, increased risk of cardiovascular diseases (such as diabetes and obesity), poor breast milk secretion, colon cancer, chronic heart diseases (CHD), among others [10]. Poor meal quality also increases the risk of micronutrient deficiency diseases of public health importance which are iron deficiency anaemia, Vit. A deficiency disorder, iodine deficiency disorder and zinc deficiency [4].

Nutrition literacy, socioeconomic status, parity, demography, affordability, and culture significantly influence the quality of a woman’s diet and overall health [6]. However, among these factors, nutrition literacy (knowledge) has a strong influence on nutrition practices regarding food acquisition, handling, preparation, processing, preservation and consumption pattern among women and their household members [11].

Adequate nutrition knowledge is significant for tackling malnutrition and various diet-related non-communicable diseases across all groups because it influences healthy food habits and adequate nutrient intake and utilization, especially among mothers [12]. However, misconstrued nutrition information can negatively impact feeding habits and health outcomes among these vulnerable groups [13, 14].

In Africa, one in every five people was hungry in 2020, and about 5 to 20% of women are undernourished, having a low body mass index (BMI) attributable to chronic hunger [3, 13]. Similarly, the Africa regional overview of food security and nutrition 2021 reported that about 29.4 million people, predominantly women with dependent children, were undernourished, while 21.4% (43 million) people were food insecure in 2020 [15]. The prevalence of anaemia among women in Africa was the highest (51.8%), while the prevalence of adult obesity was 12.8 per cent. However, among pregnant women, anaemia prevalence was 57%, which is attributed to inadequate dietary diversity and poor feeding patterns [15, 16].

In Sub-Saharan Africa, Tikuye *et al*., [17] reported that the prevalence of chronic and acute undernutrition among women was 10–20% and 20–25%, respectively. The Food and Agriculture Organization (FAO) 2021 report showed that about 18.7% of people, primarily women in the West Africa region, are poor and hungry, with about 68.3% (274 million) people being moderately or severely malnourished [15, 17]. Anaemia, Vit. A and Zn deficiencies among women in Africa range from 21 to 80%, while anaemia prevalence among women in West Africa is alarming at 51.8% [3, 13]. A study in Ghana revealed that 57% of the women were anaemic and overweight, a form of a double burden of malnutrition [3].

The trend of triple burden of malnutrition (that is, the coexistence of undernutrition, overnutrition and micronutrient deficiency within the same individual, household, community, and population) and other preventable diet-related diseases among women in low- and middle-income countries (LMICs) like Nigeria is alarming [9]. In 2016, the prevalence of overweight was 5% among adults in Nigeria [15]. In Nigeria, malnutrition among mothers has continued to rise in recent times. Asomugha *et al*., 2017 reported that about 11.6% of women suffered from chronic undernutrition, while 14.2% and 5.7% of women of childbearing age were either overweight or obese, respectively. Similarly, about 12.7% of mothers had iron deficiency anaemia in Nigeria [18]. This prevalence remains a considerable challenge [5, 13]. The national prevalence of malnutrition among women of reproductive age was 11 per cent. At the same time, overweight/obesity was 28 per cent. However, this varies across the sociodemographic and geopolitical zones within the country, with the highest prevalence of malnutrition reported in the northern and rural regions due to an increase in poverty and inflation rate [19–22].

Poor financial status, food illiteracy and overdependence on starchy staples are significant problems among many households in Nigeria, causing malnutrition [23]. Consequently, maternal malnutrition resulting from poor intake and poor dietary knowledge is a major predictor of poor health outcomes, such as maternal and child morbidity and mortality in most developing countries, especially in rural areas and among the low- and middle-income earners in urban centres across the nation [24–26]. A study conducted in south-south Nigeria revealed that the proportion of mothers who know the various food classes was low (35.0%) [27]. The outcome of poor maternal knowledge and intake is often expressed as low maternal nutritional status, low pre-pregnancy BMI and pregnancy weight gain, obesity, diabetes, coronary heart disease, hypertension, and high maternal and infant morbidity and mortality [28].

Moreover, poor dietary intake among women in Nigeria has been attributed to the high poverty rate, high food prices, high parity, and family size (especially in the northern region), inadequate nutrition knowledge, cultural food fads, seasonality, poor food systems and supply chain [18, 19, 21, 29]. Other factors are educational status, taste, convenience, income, and religious beliefs [30–34].

Additionally, the quality and sources of nutrition information among Nigerian mothers also play a significant role in the feeding pattern and health outcomes of mothers and their household members. This varies significantly across the different social classes, age groups, educational status, wealth index and demography in the country [11, 35]. Some identified sources of nutrition information are television, radio, newspapers and social media platforms, religious gatherings, family members and friends, educational books and visuals and health professionals, nutrition education interventions and campaigns, and academic workshops/institutions [8, 27, 35].

The causes of nutrition-related health problems across all age groups are widely documented, and their prevalence and influence of demography on the nutrition and health status of mothers differ from one region to another in Nigeria. In many resource-scarce communities, women’s diets are monotonous, dominated by starchy staple foods, and do not provide sufficient micronutrients. Thus, there exist vulnerabilities and gaps in micronutrient intakes among these groups, consequently, predisposing them to health vulnerability and preventable causes of diet-related mortalities [2].

Although improving dietary quality is multidimensional, promoting diverse diets through scientifically informed and accessible nutrition information is one of several tools that can help to ameliorate nutrition insecurity, malnutrition, poor health, and obstetric outcomes among mothers. However, there is scanty information on the relationship between the nutrition knowledge of mothers and their health vulnerability in Niger State, Nigeria. Therefore, this study aims to assess the nutrition knowledge of mothers of children aged 3–5 years and their health status in Niger State, North-central Nigeria.

## 2. Materials and methods

### 2.1 Study location

Niger State is the country’s largest state in the North-central geopolitical zone, with its capital in Minna. Other cities include Bida, Kontagora and Suleja. The Nupe, Gbagyi, Kamuku, Kambari, Hun-Saare, Hausa and Koro form the most numerous indigenous tribes of Niger State. The State is named after the river Niger and lies on coordinates with latitude 9° 55’50.45° N and longitude 5° 35’57.12°E, and its land span is about 76,363km^2^ (29,484 sq.m). It is bordered to the North by Zamfara State, West by Kebbi State, South by Kogi State, southwest by Kwara State, North-East by Kaduna State and southeast by the FCT. The State also has an International Boundary with the Republic of Benin. It comprises 25 Local Government Areas (LGAs) and 274 political wards with a population figure of 3,950,249 [36, 37].

### 2.2 Study population

The study participants were mothers of preschool children (3-5years) who had lived in the selected communities for at least three or more months before data collection.

### 2.3 Eligibility criteria and recruitment

#### 2.3.1 Exclusion criteria

Mothers with cardiovascular diseases and terminal ailments such as cancer, stroke, HIV/AIDS, and congenital malformation were excluded from the study.

#### 2.3.2 Inclusion criteria

All mothers of preschool children who lived in the selected LGAs and consented to participate in the study were recruited. Where there was more than one mother within the same household with preschool children (polygamous setting), the older mother was enrolled on the study because decision-making regarding meal plans and choice lies on them.

### 2.4 Study design

A cross-sectional study was conducted among the mothers of pre-school children from the selected LGAs in Niger State between February and May 2022.

### 2.5 Sample size

Four hundred and fifty mothers of preschool children were recruited. The sample size was determined using the formula: n = z^2^×p(q)/d^2^ [38], where n is the minimum calculated sample size, z is the z-score of 1.96 at a 95% confidence level, p is 48.0%, the proportion of food insecure population in rural households in North central Nigeria from the previous study [39], d is 5%, the desired level of precision and q is 1−p. 20% nonresponse rate was added because of the seclusion of women from the sight of men or strangers (purdah) practised in the study area and the high illiteracy level among the women. 11 participants were excluded in the analysis because of missing data. Therefore, data collected from four hundred and fifty participants were analysed in this study.

### 2.6 Sampling technique and procedure

A multi-stage sampling technique was used to enrol the mothers across the twenty-five LGAs in Niger State, North-central Nigeria. First, the twenty-five LGAs were stratified into various political wards. Wards from each LGA were selected by a systematic sampling method using a sampling interval K^th^ obtained by dividing the total number of women with the minimum calculated sample size (n). The first ward was selected randomly, and subsequent wards were selected at every K^th^ interval. Within the selected wards, the total number of mothers of preschool children was obtained using their registration at the health facility centres. Then, the calculated sample size was proportionally allocated to the selected wards based on the number of households with children. Mothers of pre-school children were selected from each household using a simple random sampling technique. Quantitative data on nutritional knowledge, eating habits and anthropometry indices via face-to-face interviews were collected from the mothers of the pre-school children in the selected LGAs across Niger state.

### 2.7 Data preparation and statistical analysis

The Data collected were analysed with SPSS version 27.0. Body Mass Index (BMI) and waist/hip ratio were extrapolated from the collected variables. Descriptive statistics in the form of percentages and frequencies were used to present the information on sociodemographic characteristics, BMI, waist/hip ratio and feeding patterns of the mothers in the study. Bivariate and multivariable logistic regression analyses were used to identify the factors associated with maternal nutrition status and minimum dietary diversity adequacy. All independent variables associated with the nutritional status at p < 0.05 were considered statistically significant.

### 2.8 Anthropometry

Weight in kg was obtained using the bathroom-weighing scale (OMRON BCM-500), height in m^2^ was measured using a stadiometer, and waist/hip circumference was measured using a measuring tape. BMI of the mothers and waist/hip ratio was computed.

### 2.9 Minimum dietary diversity scores for women (MDDS_W)

The minimum maternal dietary diversity score (MDDS_W) of the participants were estimated using the qualitative method of food frequency consumption questionnaire (24-hour recall and 7-day recall) which is an indicator of maternal micronutrient adequacy among the study participants [40]. Fifteen trained research assistants applied the questionnaire. The tool inquires 10 food groups which are aggregated for analysis. The 10 food groups are (1) grains, white roots and tubers, and plantains; (2) Pulses (beans, peas, and lentils); (3) Nuts and seeds; (4) Dairy; (5) Meat, poultry, and fish; (6) Eggs; (7) Dark green leafy vegetables (8) Other vitamin A-rich fruits and vegetables; (9) Other vegetables (10) Other fruits. The score is the sum of the total food groups consumed by mothers in the week out of the 10 food groups accessed in the study. Households were classified into insufficient micronutrient intake (if they consumed less than or equal to five food groups the previous day) and sufficient micronutrient intake (if they consumed five or more food groups the previous day) [40]. The MDD_W tool was used because it has been established that women of reproductive age (WRA) who consume food items from five or more of the ten food groups were more likely to consume at least one animal-source food and either pulses or nuts/seeds and food items from two or more of the fruit/vegetable food groups. The 7-day food consumption score (FCS) was calculated using a cumulative summation obtained from the multiplication of daily consumption frequency by a weighted score assigned to each food group [2, 41] The summation obtained was classified as follows; poor intake (0–21.49), borderline (21.5–35.0), and adequate intake (>35) [41, 42].

### 2.10 Nutrition knowledge

The nutritional knowledge of the mothers was assessed on 12 structured questions on the meaning of healthy diets, constituents of healthy diets, the importance of each food group to human health, symptoms of nutritional deficiency, sources of nutritional information to individual mothers and the relevance of the acquired information to individual dietary habit, the mothers’ responses were classified as either correct or incorrect from the available options. Experienced senior researchers in Human Nutrition within and outside the University of Kwazulu-Natal, South Africa, validated the questionnaires. The mother’s responses were also assessed using the Likert scale as strongly agreed = 5, agreed = 4, undecided = 3, disagreed = 2 and strongly disagreed = 1. Responses within the cut-off point of 3.5 are classified as good, while those below the cut-off point are classified as poor [43].

### 2.11 Ethical consideration

This study was reviewed and approved by the Biomedical Research Ethics Review Committee of the University of Kwazulu-Natal, South Africa (protocol number BREC/00003392/2021) and the National Health Research Committee in Nigeria (NHREC) (Approval Number; NHREC/01/01/2007-11/01/2022). Verbal informed consent and assent were also obtained from unlearned mothers. Written consent was obtained from learned mothers who volunteered to participate in the study by appending their signatures on the questionnaires after explaining the study’s objectives.

## 3. Results

### 3.1 Socioeconomic and demographic characteristics of the mothers

Four hundred and fifty mothers participated in this study; the majority (63.8%) were between 26–35 years old. Most (76.4%) mothers were rural dwellers and from the Nupe (68.4%) ethnic group (Table 1). The mean age of the mothers was 28.14 ± 6.03 years, and almost all (98.2%) were married. About one-third (45.0%) never had any formal education besides Islamic education. Most (63.6%) mothers were multiparous (Table 1). Maternal age, ethnicity, occupation, income and method of contraception were significantly associated with maternal nutrition knowledge (p<0.05) as indicated in Table 1.

**Table 1:** Association between socioeconomic and demographic characteristics of mothers and nutrition knowledge of the mothers of preschool children.

Variables	Frequency (*n* = 450)	Percentage (%)	χ^2^	*P-value*
**Age group**			81.623^a^	< .001*
15–25 years	89	19.8		
26–35 years	287	63.8		
36-45years	67	14.9		
>46 years	07	1.5		
**Marital status**			1.792^a^	0.181
Married	442	98.2		
Single	08	1.8		
**Parity**			.554^a^	.758
Primiparous	33	7.3		
Multiparous	286	63.6		
Grand multiparous	131	29.1		
**Occupation**			47.259^a^	<0.001*
Civil servant	20	4.4		
Artisan	19	4.2		
Full housewives	123	27.4		
Self-employed/ petty trading	206	45.8		
Farming	82	18.2		
**Income range**			8.812^a^	0.032*
<#33,000	377	83.8		
#33,000-#50,000	62	13.8		
#51,000-#100,000	05	1.1		
#101,000-#150,000	06	1.3		
**Residential area**			0.347^a^	0.556
Urban	106	23.6		
Rural	344	76.4		
**Source of potable water**			6.923^a^	0.074
Protected	197	43.8		
Unprotected	253	56.2		
**Method of contraception**	416	92.4	27.506^a^	<0.001*
Natural	34	7.6		
Scientific	-	-		
None	-	-		

The values in asteric are statistically significant at p<0.05

#### 3.1.1 Knowledge of balanced diet among the mothers

**Fig 1:**
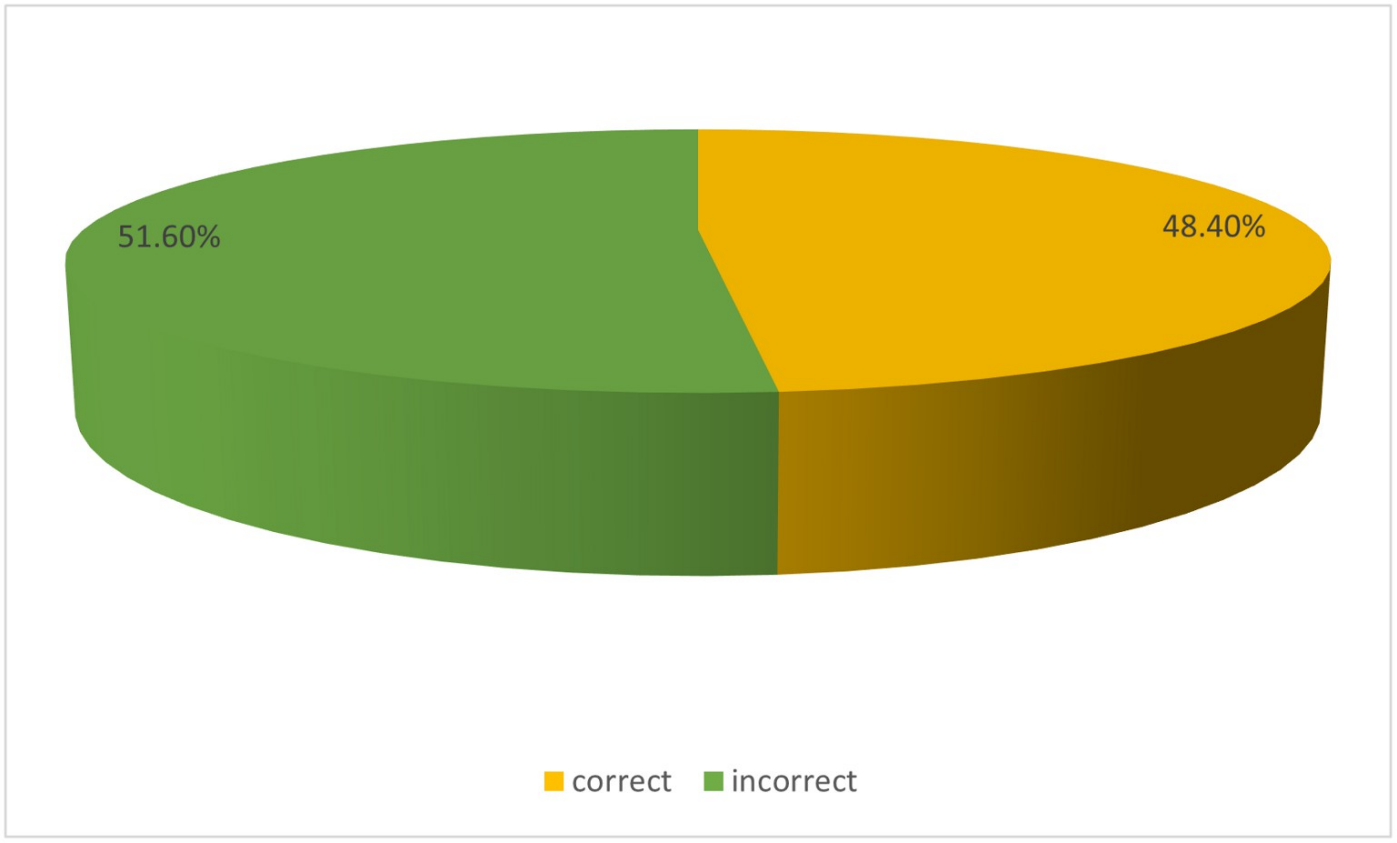
The proportion of mothers who could define what a balance diet is.

**Fig 2:**
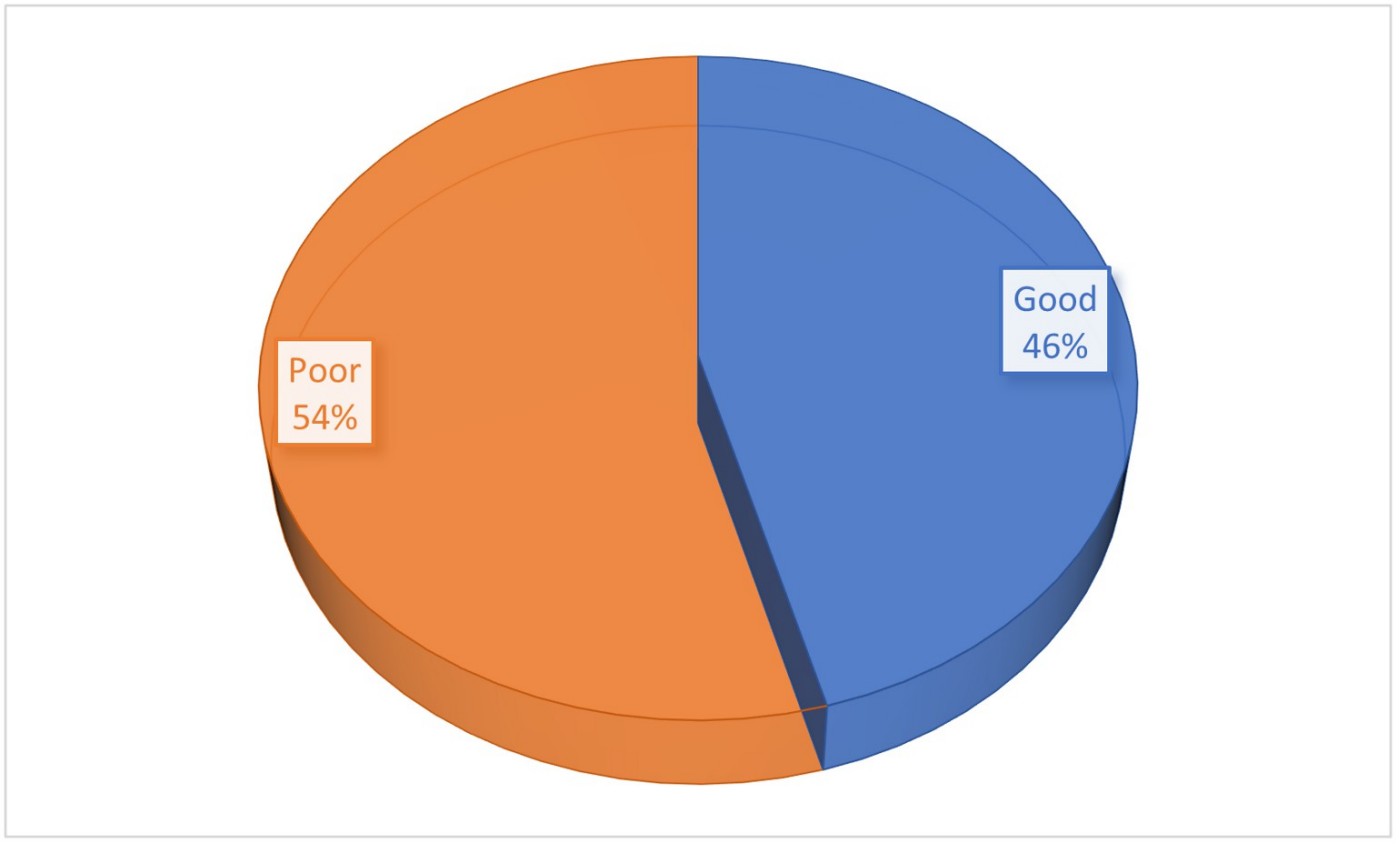
The proportion of mothers with basic nutrition knowledge score.

In Figs 1 & 2, more than half (51.6% and 54%, respectively) of the mothers had poor definitions of a balanced diet or healthy eating and lacked basic nutrition knowledge.

#### 3.1.2 Sources of nutrition information among the mothers

**Fig 3:**
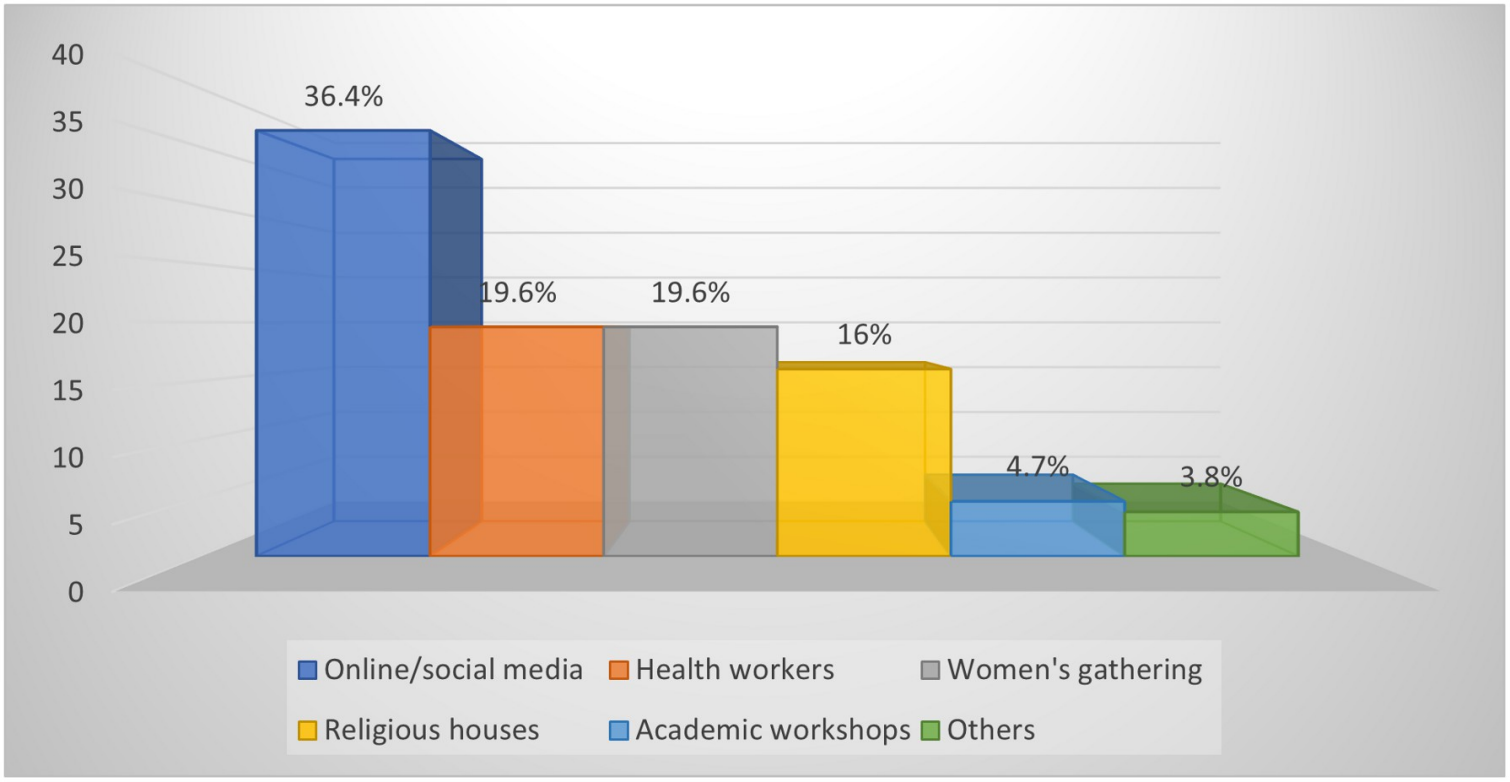
Maternal sources of nutrition information.

Among all the sources of nutrition information available to the mothers, online/social media was the most employed on nutrition issues (36.4%). Traditional media such as television, radio, billboards, and poster advertisements were the second most used source of nutrition information, while few (19.6%) mothers sought nutrition information from community health workers or community women’s gatherings. Families/relatives’ forums were the least explored platform of nutrition information among the mothers as indicated in Fig 3. About two-thirds (36.4%) of the mothers who explored social media platforms for nutrition information either had mobile phones or a relative that owned a mobile phone with a regular data subscription.

### 3.2 Meal quality of the mothers of the preschool children

#### 3.2.1 Feeding pattern, consumption pattern and minimum dietary diversity of the mothers

**Fig 4:**
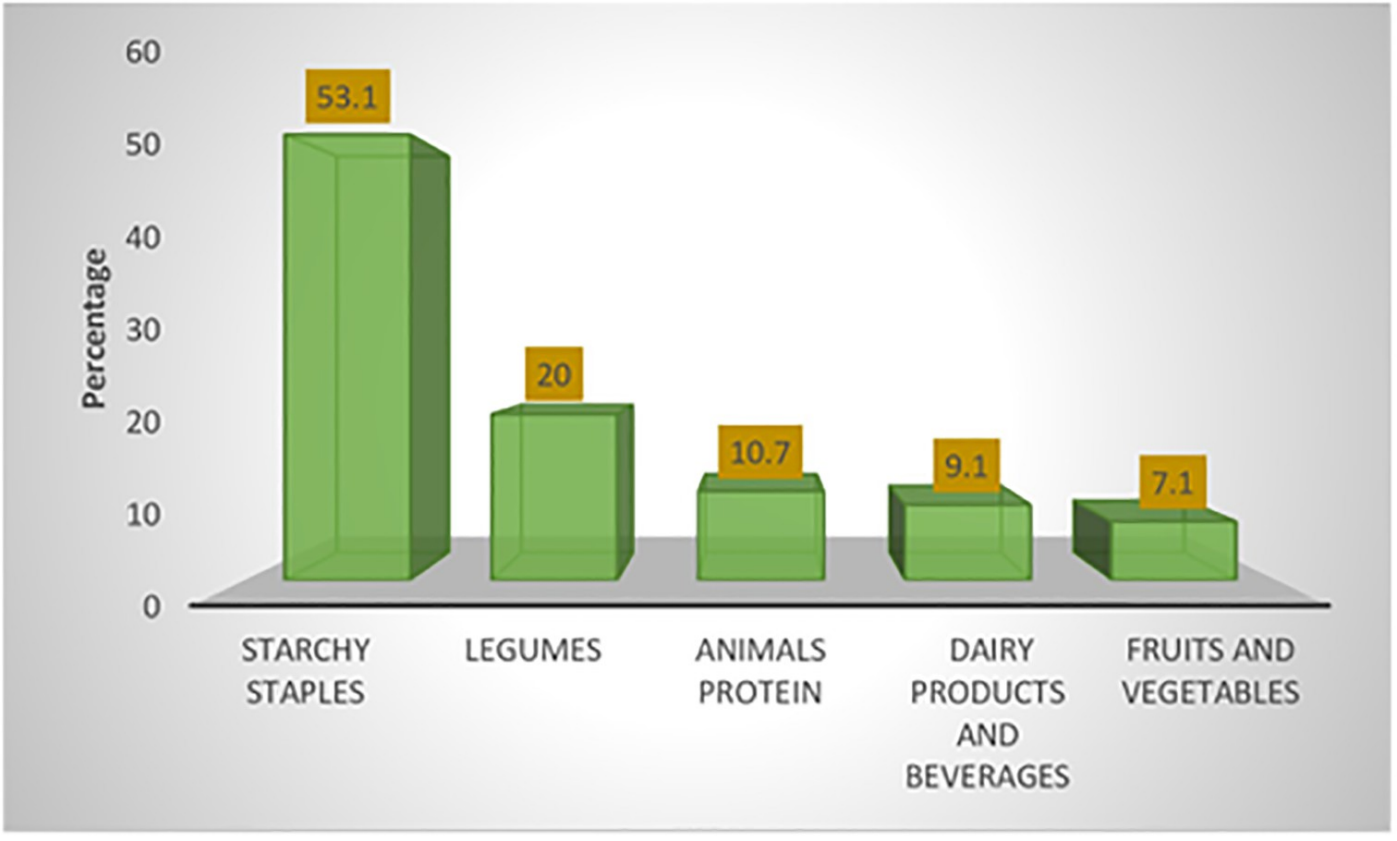
The most consumed food group among the mothers.

The frequency and weekly consumption pattern of the various food groups among the mothers based on 7-day dietary recall was captured in Table 2 and Fig 4. Table 2 showed that about 62.9% of mothers consume starchy staples between three to six times daily. In comparison, only 26.9% of the mothers had sweets and snacks between three to six times daily. These starchy staples usually contain rice and fermented beverages such as “pap” and ’Kunu’ from millet grains. Fruits, vegetables, and dairy products were the least consumed food groups (7.1% and 9.1%, respectively), as indicated in Fig 4.

**Table 2:** Weekly food consumption pattern for mothers.

Food Groups	NEVER		YES					
	*N*	%	Once to twice weekly		Thrice to Six times		Total Yes	
			*N*	%	*N*	%	*N = 450*	%
Starchy staples	19	4.2	148	32.9	283	62.9	431	95.8
Dark green vegetable	39	8.7	244	54.2	167	37.1	411	91.3
Vit. A fruit and Vegetable	98	21.8	273	60.7	79	17.6	352	78.2
Offal and Organ consumption	100	22.2	264	58.7	86	19.1	350	77.8
Other fruits and Vegetable	88	19.6	291	64.7	71	15.8	362	80.4
Meat and Fish consumption	56	12.4	229	50.9	165	36.7	394	87.6
Egg consumption	101	22.4	282	62.7	67	14.9	349	77.6
Legumes, nuts, and Seed consumption	40	8.9	211	46.9	199	44.2	410	91.1
Milk and milk derivatives	100	22.2	260	57.8	90	20.0	350	77.8

**Fig 5:**
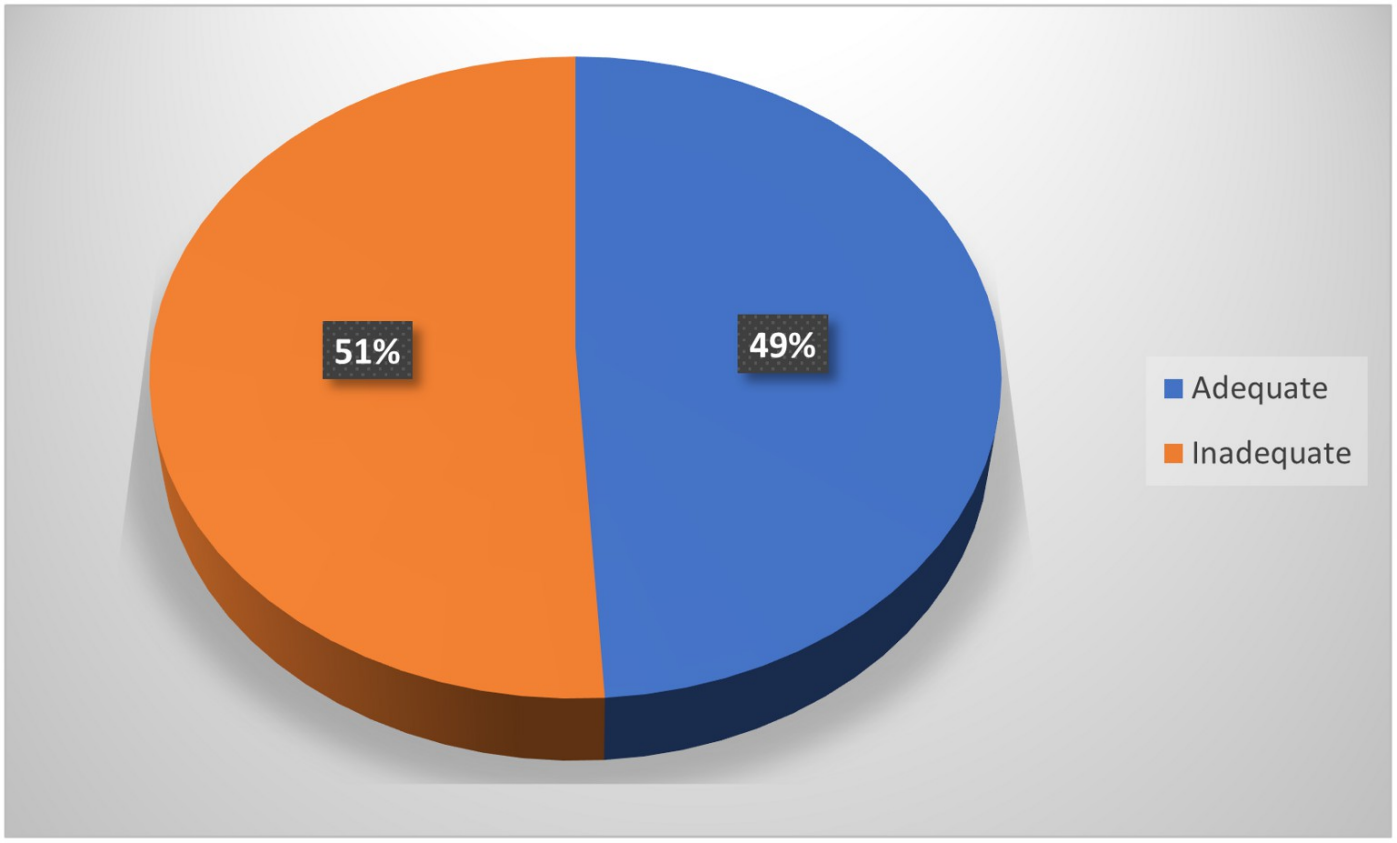
Minimum dietary diversity of the mothers based on 24-hour recall.

**Fig 6:**
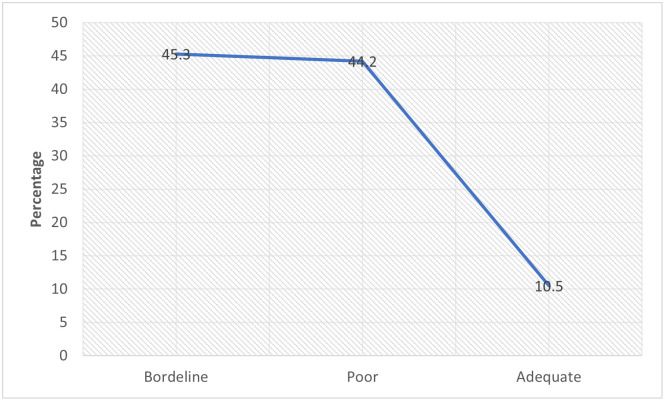
Seven-day food consumption score (FCS) of the mothers.

**Fig 7:**
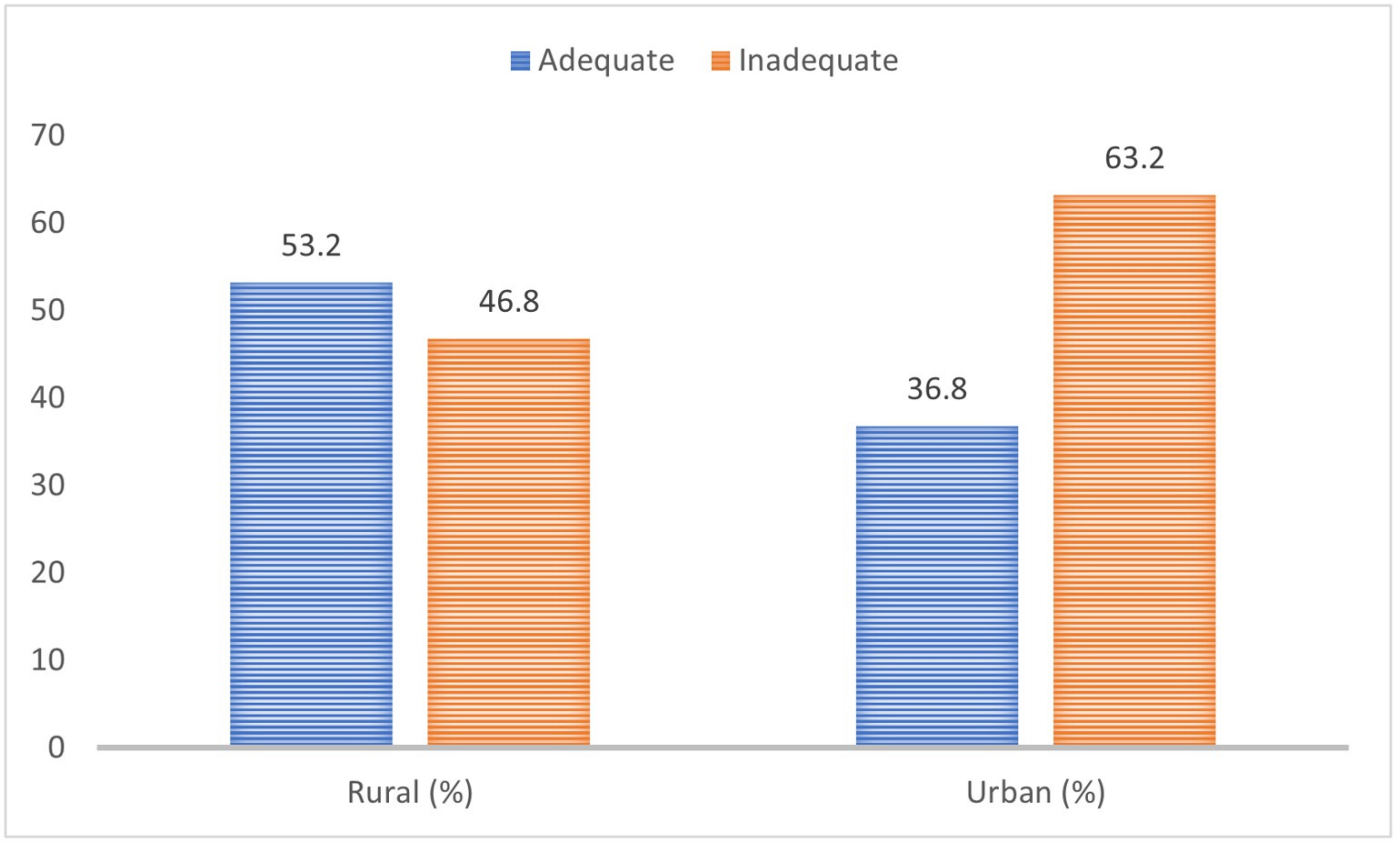
Minimum dietary diversity of the mothers based on the residential area.

Using the 24-hour dietary recall, about half (50.7%) of mothers did not meet up with their daily minimum dietary diversity (MDDS_W) as recommended by FAO, FHI 360 [40], while the 7-day food consumption score (FCS) revealed that only 10.5% of the mothers met their weekly food consumption score adequately as indicated in Figs 5 & 6 respectively. Fig 7 shows the proportion of mothers who met the minimum dietary diversity based on their place of residence using the 24-hour dietary recall.

#### 3.2.2 Impact of maternal nutrition knowledge on meal quality and food acquisition

**Fig 8:**
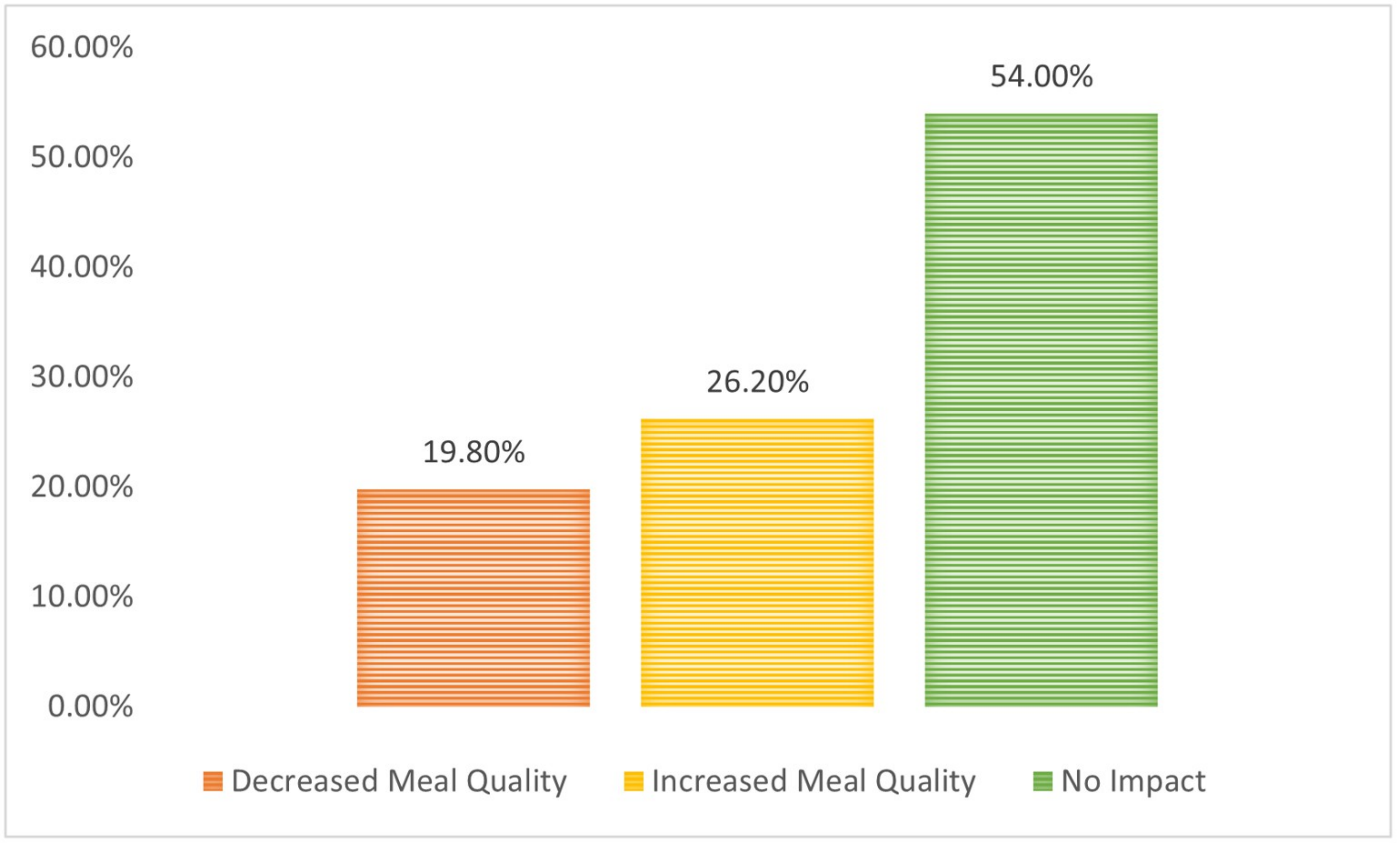
The proportion of mothers whose nutrition knowledge impacted their meal quality.

More than half (54.0%) of mothers in the study indicated that the nutrition knowledge acquired did not impact the quality of meals consumed or their food choices/preferences (Fig 8).

### 3.3 Anthropometric status of mothers of the preschool children

**Fig 9:**
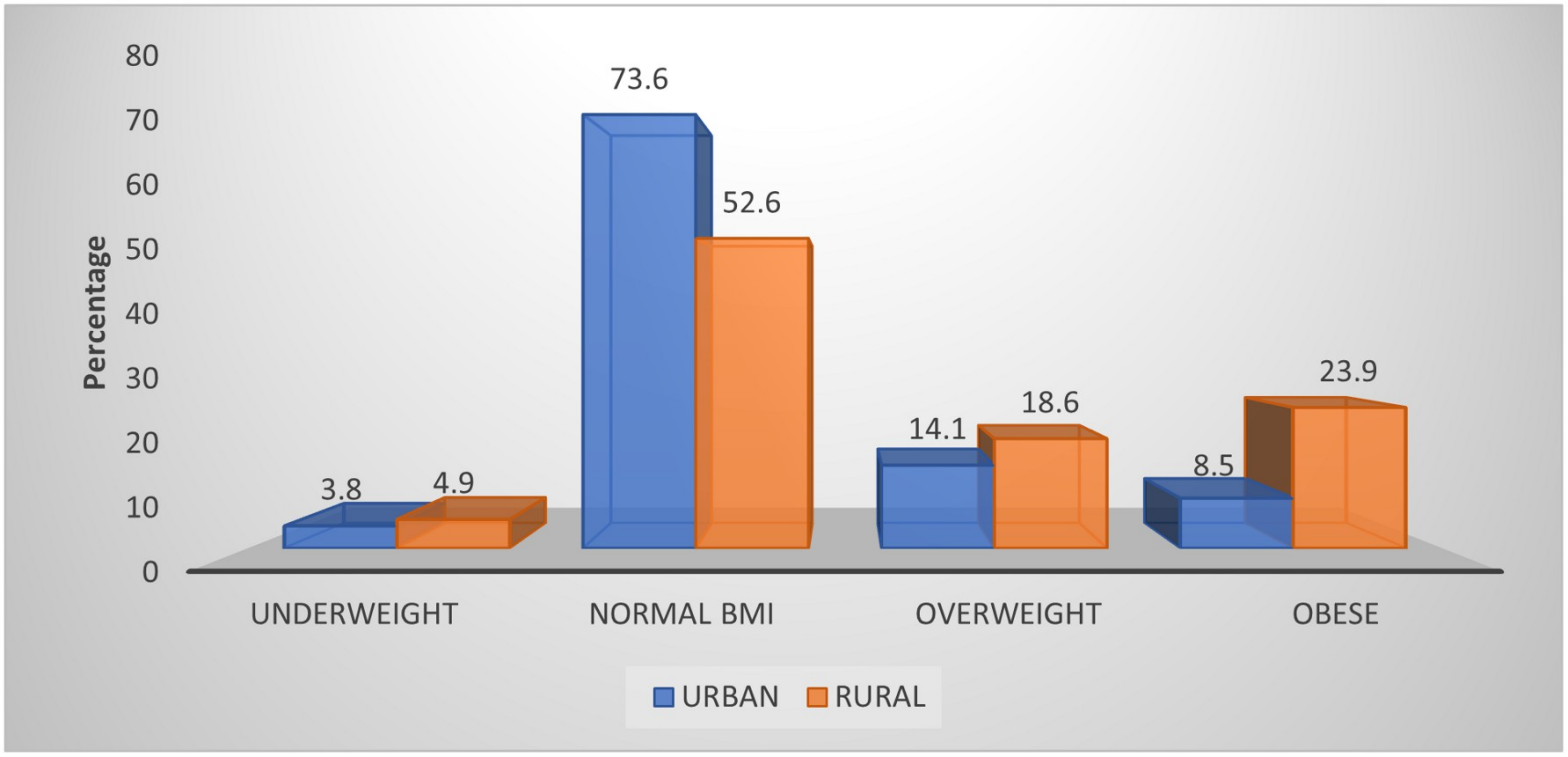
Body mass index of the mothers based on the residential area.

The anthropometric indicator of the mothers is presented in Fig 9 and Table 3. More than half (57.6%) of the mothers were within the normal range (18.5kg/m^2^–24.5kg/m^2^). The mean maternal BMI and waist/hip ratios were 24.52 ± 5.31 kg/m^2^ and 0.82 ± 0.08, respectively. However, their waist/hip ratio showed that the majority (58.9%) had a value less than 0.80, at a low risk of developing cardiovascular diseases.

**Table 3:** Risk of developing coronary heart disease among the mothers.

Waist/hip ratio range	Risk of CHD	Frequency (*N* = 450)	Percentage (%)
≥0.86	High health risk	135	30
0.81–0.85	Moderate health risk	54	12
≤0.80	Low health risk	261	58

### 3.4 Association between sociodemographic characteristics and minimum dietary diversity of the mothers

The linear regression model showed that ethnic group, occupation, residential area, and method of waste disposal were risk factors for inadequacy in the minimum dietary diversity among the mothers (Table 4). The body mass index (BMI) of the mothers was significantly associated with and a predictor of the adequacy of their minimum dietary diversity (*p* = 0.000, unstandardised β = 24.358, F value = 0.064, 95% CI = 23.011–25.706). However, there was no association between maternal nutrition knowledge and their minimum dietary diversity (*p =* 0.09, χ^2^ = 13.682).

**Table 4:** The association between sociodemographic characteristics and adequacy of minimum dietary diversity score using a regression model.

**Variables**	**χ** ^**2**^		***P-value***
Residential area	8.725		0.003*
Occupation	20.439		0.005*
Source of potable water	7.685		0.053
Method of waste disposal	9.228		0.026*
Maternal nutrition knowledge	13.682		0.090
**F-value**	**R**	**Unstandardized β**	**95% CI**
1.666	0.136	4.212	3.163–5.260

The asterisk values were statistically significant at *p*<0.05.

## 4. Discussion

This study assessed mothers’ sociodemographic and economic status, nutrition knowledge and nutritional status in selected LGAs of Niger State, North-central, Nigeria. More than two-thirds of the mothers were 26–35 years old. It is consistent with a study conducted among lactating mothers by Tessema *et al*., [1] and Kibre *et al*., [44], where the majority (71.0% and 49.0%, respectively) of the mothers were more than 25 years of age. It is, however in contrast to the findings of Hundera *et al*., [14] and Ajisha *et al*., [45] where the majority (81.3% and 57.5%) of the mothers from a study conducted in Ethiopia and India, respectively, were below 25 years. Studies have reported a positive association between maternal age and nutrition knowledge, maternal educational status and healthy eating habits [10, 27, 28] study also show a positive association between maternal age and nutrition knowledge (*p*<0.05) (Table 1). More than two-thirds of the mothers were Nupes. This could be because Nupe is the major tribe in the study area. Most of the mothers were married. The high proportion of married participants could be attributed to Islamic practices encouraging early marriages. Also, poor disposition to female education and poverty predisposes the girl-child to be married off early. This practice is common among poor-income and uneducated households in the northern regions of Nigeria [46]. The finding agrees with an Ethiopia study by Kibre *et al*. [44] who reported a similar trend of a high proportion of married women. In contrast, a study in Ghana showed that almost all (91.0%) of the mothers were not legally married [10].

Less than half (45.8%) of mothers in this study were petty traders which is consistent with the findings of Appiah *et al*., [10] where 45.2% of the mothers were petty traders. It could be because most of the mothers were from rural communities where farming is the main occupation of their husbands. However, it contrasts with the findings of Kibre *et al*., [44] and Smith *et al*., [47] where about 70% and 49.0% of the mothers were farmers, respectively. Also, Ajisha *et al*., [45] reported that most mothers in their study area were full housewives. Maternal occupational status was strongly associated with nutrition knowledge (*p* = <0.001; χ^2^ = 47.259). However, poor maternal occupational status contributes significantly to the low income among mothers, thus, affecting their ability to afford nutritious food or meet their minimum dietary diversity for healthier living and productivity [27, 28].

Above half of the mothers had no formal education besides Islamic education. This high proportion of non-formal education may be due to poor enrolment of females in schools, also, early marriage, pregnancy, and childbirth which are predominant in Northern Nigeria might have led to the termination of education earlier than expected [27, 28]. This finding contrasts with the report of Kibre *et al*., [44] and Ajisha *et al*., [45] where most mothers had primary education. It is also in contrast to the study by Smith *et al*., [47] who reported that a higher proportion of mothers had secondary education. Educational status influences maternal nutrition knowledge, food acquisition and maternal dietary quality [27, 28, 48]. However, poor educational status in this study may have contributed to the low maternal nutrition knowledge and poor dietary diversity seen. Maternal illiteracy, therefore, is a potential risk factor for poor dietary quality, diet-related morbidity, and mortality among mothers in most developing nations at large [1, 27].

Most mothers in the study area were rural dwellers which may be because Niger state has more rural settlements than urban settlements. This finding is similar to the report of Kibre *et al*., [44] where most mothers resided in rural areas; however, it is in contrast with the findings of Karcz *et al*., [49] and Ajisha *et al*., [45] where most of the mothers resided in the city. Most (53.2%) of the mothers from rural communities could meet their minimum dietary diversity cut-off than mothers (36.8%) from urban communities. Hence, residential areas played a vital role in meal quality among the mothers (Fig 7). This could be because mothers in rural communities tend to have more access to varieties of food at lower prices, and many also acquire food items from their farm produce or as a transfer/gift from fellow farming households than the urban community dwellers who patronise standard malls and grocery shops at a higher cost. Secondly, rural dwellers are more likely to consume more varieties of indigenous staples, fruits and vegetables from vast harvests and less processed foods than urban dwellers due to proximity to local produce markets, ownership of home gardens, and farmlands [50]. Other factors are the affordability of such food items and the cheaper cost of living in rural communities than people in the city centres. However, factors that influence the minimum dietary diversity among the mothers were residential area, occupation, and method of waste disposal (p,0.05) (Table 4) and consistent with previous findings [51–53].

Above half of mothers could not define what a balanced diet/healthy eating is, and they also showed poor nutrition knowledge, It, however, contrasts with the findings of Hundera *et al*., [14] where more than two-thirds of the mothers could define what a balanced diet is; also, less than half of the mothers had good basic nutrition knowledge. This finding could be due to the poor formal educational status among the mothers since basic nutrition information is taught in formal schools. A similar study among young mothers in Ghana by Appiah *et al*., [10] also showed that less than half (44.9%) of the women had good nutrition knowledge. The current study contrasts with Hundera *et al*., [14], who reported that more than half (58.3%) of nursing mothers in Ethiopia had good nutrition knowledge. Maternal nutrition knowledge has been reported to influence the level of feeding patterns, nutritional status, and healthy lifestyle practices among women in previous studies [27, 28, 54] However, more than half (54%) of the mothers in this study indicated that nutrition knowledge never improved their meal quality. Also, there was no statistical association between maternal nutrition knowledge and education and dietary quality (*p* = 0.09; χ^2^ = 13.682), this is consistent with Audu [55] in a similar study reported where there was no significant association between maternal education and nutrition attitude of women. In contrast, Sawadogo et al. [54] reported that maternal educational level positively impacts the quality of nutrition knowledge, dietary practices, and health-seeking behaviour of mothers, significantly impacting the health outcomes of mothers and their under-five children. The reason why there is no statistical association between maternal education and dietary quality in this study could be the influence of other underlying factors on meal quality, such as poor food environment, seasonality, the impact of climate change on food production, cultural food preference, the high cost of healthy, nutritious, and safe foods as compared to the affordability of convenient and ultra-processed foods and, most recently, the negative impact of insurgency and Covid-19 pandemic on food security across the nation [56]. However, Vaitkeviciute *et al*., [57] and Jemide *et al*., [27] reported a positive impact of nutrition knowledge on meal quality.

Among the sources of nutrition information available to the mothers, online/social media was the most patronised for information on nutrition issues (36.4%) because most of the mothers either had a phone or a relation that had a mobile phone in the family, also data subscription is available in several pocket-friendly forms by most service providers either as day, night, weekly or monthly basis. In a similar study by Quaidoo *et al*., [8] in Accra, Ghana, most (78.1%) participants also employed online/social media as their source of nutrition information. However, in contrast, Tessema *et al*., [1] reported that 63.6% of mothers in their study acquired nutrition information from health workers. The wide use of online/social media platforms for nutrition information in this study could be due to high access to mobile phones and an increase in telecommunication coverage in many rural communities across Nigeria [58, 59]; however, this information did not impact positively on maternal meal quality in the study area. Secondly, most mothers in this study were within the young adult age group (26–35 years). Hence, there exists a higher tendency among the younger generation to assess online resources for information on various subject matter including nutrition at the convenience of their home [60] more than the older generation due to availability of numerous pocket-friendly services providers and mobile facilities readily affordable. However, information shared on social media must follow ethical procedures and professionalism to avoid misconstrued content by the public [61, 62].

It is noteworthy, that most of the nutrition information retrieved from these platforms is based on foreign food items and might not be from qualified professionals [63, 64], thereby promoting unhealthy feeding practices among low-income earners who cannot afford them yet neglect available indigenous nutrient-dense staples which are tagged ‘food for the poor’ [65]. Also, most of the information is usually void of indigenous knowledge systems (IKS) therefore, it is not adaptable to local contents of the utilization of neglected nutrient-dense staples, and vegetables which are readily available in many localities but unexplored or well consumed [34, 55, 66]. It has been reported that to improve the food security and health status of indigenous peoples, there must be the promotion of cultural strength and inclusion of traditional food systems [67, 68]. Another possible reason could be poor socio-cultural acceptance of most recommended healthy foods via social media bloggers due to food fads/taboos, and lack of incorporation of indigenous food crops native to African descent [32, 33].

Although most mothers explored social media for nutrition information, many needed to understand what a balanced diet entails and needed to have basic nutrition knowledge of the six food groups, their health benefits, and deficiency symptoms of lack of essential nutrients. Many do not know how to prepare and lack indigenous knowledge on the sources and the right proportion to consume from each food group for a healthy life. This could be because most of the information about food among mothers is age-long information often passed down from generation to generation by mothers and grandmothers, especially in many rural communities across Africa [55, 63]. Also, the poor educational status of the mothers could contribute to the poor knowledge/awareness and poor food choices [69] since the majority did not have formal education where basics of food and nutrition information are taught as home economics subjects from the universal basic education (UBE), which is, primary school through secondary school education across Nigeria [69]. It has been reported that well-taught nutrition education courses targeted at women could help mothers to be well-motivated, knowledgeable, skilled, and empowered to make informed choices on nutritional related-issues, healthier food choices and improve the health-seeking behaviours for themselves and their households [34, 55, 69].

Sources of nutrition information and the target audience greatly determine the credibility and receptibility of such information. This is because nutrition information from sources such as online platforms has been reported not to contain local content or indigenous food for rural dwellers and middle- and low-class citizens to incorporate readily in meals [66]. Secondly, most of the information in the media promotes westernised food above indigenous nutrient-dense diets which are often not pocket-friendly, hence such food environments or food systems are not sustainable for enhancing nutrition security and optimal health outcomes among women from resource-limited households [70]. Similarly, the credibility of social medial sources of nutrition information is less specific and beneficial, especially when they are not from a professional and scientific standpoint. Although, Quaidoo *et al*., [8] in a similar study conducted among young adults in Accra, Ghana, reported that the participants ascertained the credibility and reliability of online sources of nutrition information above other sources. In contrast, Lee *et al*., [71] reported that nutrition information obtained from professionals (clinicians and midwives) is more dependable and reliable. It is imperative to note that adequate nutritional knowledge and appropriate healthy practices play a significant role in determining optimal health outcomes for mothers. Therefore, providing quality nutritional information via an indigenous knowledge system (IKS) to all mothers is a prerequisite for reducing diet-related maternal morbidity and mortality in many developing countries [1, 27]. Traditional media such as television, radio, billboards, and poster advertisements were the second most used source of nutrition information which is in contrast with the report of Sawadogo et al., [54] where traditional media was reported as the most explored source of nutrition information. A few (19.6%) mothers sought nutrition information from either community health workers or community women’s gatherings, respectively. Families/relatives and academic forums were the most petite medium of nutrition information among the mothers in this study. This could be because more people are increasingly accessing internet services via portable digital devices like mobile phones, mast/towers, fibre optics, Ipads, and Wi-Fi in homes, offices, malls, recreational and educational centres, and other public places [58, 59]. Moreso, online sources are relatively cheap and more convenient than consulting a few costly community health workers, professional nutritionists, and dietitians in various healthcare facilities across the nation. Also, information on social media is cost-effective for companies, individuals, government, and non-governmental organizations coupled with the wider coverage it has hence, the paradigm shifts from the traditional medial platforms. This implies that people do not necessarily have to be formally educated before they are informed or enlightened on several health-related issues due to the evolution of several online resources like animations, wordless short-video clips that have made the universe a global village and information ubiquitous [72, 73].

Starchy staples were the most consumed food among the mothers, and these usually consisted of staples like rice and fermented beverages (such as pap and ’Kunu’) from millet. Fruits, vegetables, and dairy products were the least consumed food groups (7.1% and 9.1% respectively). It is similar to the finding of Appiah *et al*., [10] who reported that the starchy/cereal group was the most consumed food group per day while fruits and vegetables were the least consumed among their participants. The high consumption of starchy staples “full but empty plate” among the mothers in the current study could be because Niger State is one of Nigeria’s primary producers of cereal crops since, its lies in Guinea Savanna (middle belt) zone [74]. The low consumption of fruits and vegetables may be due to the lack of or poor knowledge of their benefits, poor availability due to seasonality or lack of financial means to purchase them [75–79]. Animal proteins (such as offal, beef, and poultry products) and dairy products were also among the least consumed food groups. This complements the findings of Appiah *et al*., [10]. However, animal protein consumption could be low due to costs and financial constraints, while food taboos, cultural beliefs, and myths regarding their consumption among nursing mothers could be a factor.

About half (50.7%) of the mothers needed to meet up with the minimum dietary diversity for women (MDDS_W) as recommended by the Food and Agriculture Organisation [2, 40]. The low proportion of mothers who met their dietary diversity based on the seven-day food consumption score in this study (Fig 6) could be attributed to factors such as poor financial and empowerment, poor educational status, occupational status and over-dependence and monotonous consumption of readily accessible starchy staples. Other factors, reported by Katenga-Kaunda *et al*., [80] could be the lack of possession of a home garden, small-scale livestock and animal husbandry, food myths based on cultural and religious beliefs, food illiteracy and poor nutrition knowledge of the health benefits of essential food groups. However, for mothers that met their minimum dietary diversity, the adequacy of micronutrient intake may not be ascertained since adequacy depends largely on the quantity of micronutrient-dense food consumed [40].

BMI is an indicator of body size and composition; it helps predict nutritional status. However, the waist-hip ratio was additionally used because it is superior to BMI in predicting cardiovascular disease (CVD) risk [81, 82]. Waist/hip ratio measures abdominal adiposity and coronary heart disease (CHD) risk. This is because increased visceral adipose tissue is associated with metabolic abnormalities, and myocardial infarction (MI), including decreased glucose tolerance, reduced insulin sensitivity and adverse lipid profiles, risk factors for type -2 diabetes and deaths [81]. The recommended range for women is 0.85 or below; the higher the value, the higher the risk of CHD in women [82].

The BMI of the mothers in this study indicated that more than half (57.6%) of the mothers were within the normal range of 18.5kg/m^2^–24.5kg/m^2^, few (17.6%, 4.7%) of the mothers were obese or underweight respectively. It contrasts with a study conducted among women of reproductive age in Cameroon by M’bobda *et al*., [83] where most mothers were either overweight or obese (30.8%, 38.5%), respectively. A study in Lesotho by Rothman *et al*., [84] also reported a higher proportion of obesity among women. The mean maternal BMI and waist/hip ratio were 24.52±5.31 kg/m^2^ and 0.82± 0.08, respectively. However, their waist/hip ratio showed that the majority (58.0%) had a value less than 0.8 (Table 3) which indicates a lower risk of developing coronary heart disease (CHD). The proportion of obesity was higher among rural women (23.9%) than urban women (8.5%). It is similar to the findings of Trivedi *et al*., [85] who reported a high level of obesity among rural women than urban women; however, this finding contrasts with the findings of Rothman *et al*., [84] where obesity was higher among urban women than the rural women. the high proportion of obesity among rural women could be attributed to their occupational status and sedentary lifestyles, as most were petty traders who sold their goods within their residential area.

### 4.1 Limitation of the study

The study did not assess the sufficiency of each nutrient consumed by the mothers compared with the recommended nutrient intake (RNI). Therefore, the prevalence of micronutrient deficiency among mothers could not be investigated.

## 5. Conclusion

This study explored the impact of maternal nutrition knowledge on the health vulnerability of mothers of preschool children in Niger state, North-central Nigeria and thus contributed to scanty literature in this area. Findings from the study revealed that about half of the mothers had poor nutrition education. Among the few mothers with good nutrition knowledge, there was no significant impact on their meal quality, because of several underlying factors like poverty, cultural preference, unverified nutrition information sources and poor accessibility (security threats) to healthy foods nationwide. The mothers’ poor literacy level and nutrition knowledge could also be attributed to the high informal education among mothers in the study area. Similarly, the mothers’ socioeconomic status played a significant role in the adequacy of their nutrition knowledge and validation of their information sources. Although social media was the mothers’ most explored source of nutrition information, there was no correlation between nutrition knowledge and the meal quality of the mothers. The dietary diversity of the mothers varied across their residential settings. Mothers from rural communities appeared to be more diverse in their dietary intake than mothers from urban centres; however, the quantity consumed was below most mother’s recommended minimum dietary diversity score. Although, the variation in consumption patterns could be due to the cheaper cost of living in rural environments and proximity to producing (local/farmers) markets than in urban settlements. The anthropometric indicators showed that most mothers were within the normal BMI range, while the waist/hip ratio showed a lower risk of CHDs among the mothers. However, the risk of micronutrient deficiency appears to be higher among the participants because only a few (10.5%) mothers adequately met their minimum dietary diversity score based on the 7-day food consumption score (FCS).

This study implies that efforts towards optimizing maternal dietary quality and health outcomes in Africa and other low-and middle-income countries like Nigeria must address wholistically the underlying and intermediate determinants of maternal nutrition as spelt out by the 2020 UNICEF conceptual framework of maternal and child nutrition if the Sustainable development goals 2030 and Africa agenda 2063 is to be realized.

Further studies should explore for better understanding, the impact of social media on nutrition education in north-central Nigeria and review in more detail the lack of correlation between nutrition education and the quality of the meal of vulnerable members of the households to ensure better participatory and effective implementation of community-based programs.
